# Data-driven medical devices and the EU MDR: mapping gaps in standards for regulatory compliance

**DOI:** 10.1038/s44401-026-00075-2

**Published:** 2026-03-09

**Authors:** Ugur Ilker Atmaca, Saif Ul Islam, Gregory Epiphaniou, Anita Khadka, Stuart Harrison, Nikolaos Matragkas, Scott Hansen, Rance DeLong, Carsten Maple

**Affiliations:** 1 WMG, University of Warwick, Coventry, UK; 2 Abdullah Gul University, Kayseri, Turkey; 3 CEA, List, Université Paris-Saclay, 2 Bd Thomas Gobert, Palaiseau, France; 4 The Open Group, Brussels, Belgium; 5 The Alan Turing Institute, London, UK

**Keywords:** Computational biology and bioinformatics, Engineering, Health care, Medical research

## Abstract

Data-driven medical devices promise significant enhancements to diagnostic accuracy, personalised treatments, real-time patient monitoring, and clinical decision support. However, these innovations present regulatory challenges. This study maps the current European Union Medical Device Regulation requirements against international data safety and quality assurance standards, identifying critical gaps. It highlights challenges in data quality, algorithm transparency, risk management, continuous validation, and synthetic data use. Recommendations are proposed to support regulatory adaptation.

## Introduction

Rapid technological advancements have substantially expanded both the availability and capabilities of Artificial Intelligence (AI) in healthcare. Data-driven algorithms are increasingly integrated into medical devices, where they assist in clinical decision-making, enhance diagnostic accuracy, personalise treatment strategies, and enable real-time patient monitoring^1,2^. This study defines “data-driven medical devices" as devices whose function depends on training data and runtime input data, rather than static rule-based logic, highlighting the role of data quality and assurance in regulation. This definition includes the “machine learning-enabled medical devices" defined by the International Medical Device Regulators Forum (IMDRF)^3^. However, the dynamic and continuously evolving nature of AI presents significant regulatory challenges^4,5^, including the framework of the European Union’s Medical Device Regulation (EU MDR)^6^.

Existing medical device regulations are mainly designed for conventional medical devices, and they may not fully address the challenges of data-driven medical devices^7,8^. These challenges include the lack of guidance on continuous validation of adaptive algorithms, unclear risk classification criteria for AI systems, and insufficient provisions for dataset quality and bias management^9^. Yet, there are global efforts from regulatory bodies in various jurisdictions, including the US, Japan, and the UK, and coordinated initiatives under the IMDRF to address these complexities^10–14^.

This study aims to identify gaps between the medical device requirements and the current data safety and quality assurance standards for data-driven technologies to support their use in improving regulatory processes. While prior reviews analysed the regulatory implications of artificial intelligence in medical devices, these studies mainly focused on broad-level regulatory challenges but not on the role of synthetic data in regulatory compliance and validation processes^4,15^. Building on the existing research, this study provides the following key contributions:

- It maps the EU MDR requirements to the medical device regulatory lifecycle.
- It reviews existing data safety and quality assurance standards and guidelines to identify their gaps in meeting EU MDR requirements.
- It identifies emerging challenges for the use of synthetic data in compliance and evidence generation, and it provides a set of recommendations.

## Search strategy and selection criteria

**Fig. 1:**
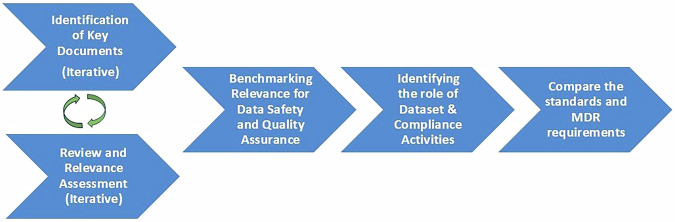
Methodology flowchart for the regulatory gap analysis of data-driven medical devices. This diagram provides an overview of the steps of the methodology to identify gaps between existing data safety and quality assurance standards and the European Union (EU) Medical Device Regulation (MDR) requirements. The process starts on the left with two iterative cycles, which are highlighted by circular green arrows connecting them: 1) `Identification of Key Documents' and 2) `Review and Relevance Assessment' for refinement of the list of materials. Once key documents are identified and reviewed, the process moves to the right through three distinct phases. First is `Benchmarking Relevance for Data Safety and Quality Assurance', where the selected documents are evaluated against safety and quality criteria. The next step is `Identifying the role of Dataset & Compliance Activities', which involves mapping data quality-related dataset characteristics to regulatory requirements. The final stage is `Compare the standards and MDR requirements', where the actual gap analysis is done.

This study reviewed academic literature and standards to identify documents relevant to data safety, quality, and compliance in the context of the EU MDR. The review focused on documents published between 2015 and 2025 to capture regulatory developments before and after the formal enforcement of the EU MDR in 2021. The remainder of the study comprised two major tasks. First, it developed a corpus of standards and guidance documents through the BSI British Standards Online (BSOL), the IEEE Standards Association Library, the IMDRF digital library, and relevant European Commission repositories. Document relevance was determined through an iterative screening process with expert judgement. This process collected 76 standards and guidance documents, which are catalogued in Supplementary Material 2. Second, a review of the academic literature is conducted using IEEE Xplore, Google Scholar, and Scopus advanced search. Search strategies combined terms such as “data quality,” “medical device regulation,” “compliance,” “digital health,” “evidence generation,” and “synthetic health data”. Records were excluded if they were non-medical, non-regulatory, outside the European context, or centred on topics beyond the scope of this review, such as “adversarial networks” or “blockchain”. The methodology, summarised in Fig. 1, steps 1 and 2, involves repeated cycles of identifying and reviewing key documents to ensure relevance and completeness. Subsequent steps benchmark these documents against EU MDR criteria, map relevant compliance activities, and conduct a structured gap analysis.

## Review of EU MDR requirements

The concept of “intended clinical benefit” has gained regulatory importance under the EU MDR, requiring manufacturers to clearly define and demonstrate their devices’ benefits during approval and through Post-market surveillance (PMS). Clinical investigations must be designed to demonstrate safety and effectiveness, supported by relevant data. Furthermore, Post-market clinical follow-up (PMCF) ensures ongoing monitoring to verify continued safety and performance. Real-world evidence collected via PMS is essential, as it may uncover risks not seen in pre-market trials, ensuring device efficacy and safety across diverse patient populations^16^.

To ensure these benefits are consistently achieved, recent regulatory changes have strengthened the role of notified bodies. These bodies are now required to conduct regular, thorough assessments of manufacturers’ compliance, including unannounced audits and tests throughout the device lifecycle. The regulations also clarify the responsibilities of economic operators such as distributors and importers, who must ensure device conformity, maintain detailed documentation, and support transparent communication across the supply chain.

Compliance with these requirements is supported by harmonised standards and guidance documents. ISO 13485^17^ provides the foundational quality management framework covering design, development, and manufacturing, while ISO 14971^18^ details the application of risk management. These are further aligned with the EU MDR through documents such as ISO/TR 20416^19^, which offers specific guidance on PMS. To address regulatory convergence across jurisdictions, the International Medical Device Regulators Forum (IMDRF) provides frameworks such as IMDRF GRRP-WG/N47^20^, which harmonises risk management and clinical evaluation approaches, thereby reducing procedural complexity.

Manufacturers often rely on interpretative documents to connect high-level regulatory requirements with practical implementation. The Medical Device Coordination Group (MDCG) documents and the Medical Device Guidelines (MEDDEV) series are pivotal in this regard. For instance, MEDDEV 2.7/1, Rev. 4^21^ remains a key reference for defining the collection, assessment, and analysis of clinical data. Together, these standards and guidelines ensure that manufacturers can generate the objective evidence required for the General Safety and Performance Requirements (GSPR).

**Fig. 2:**
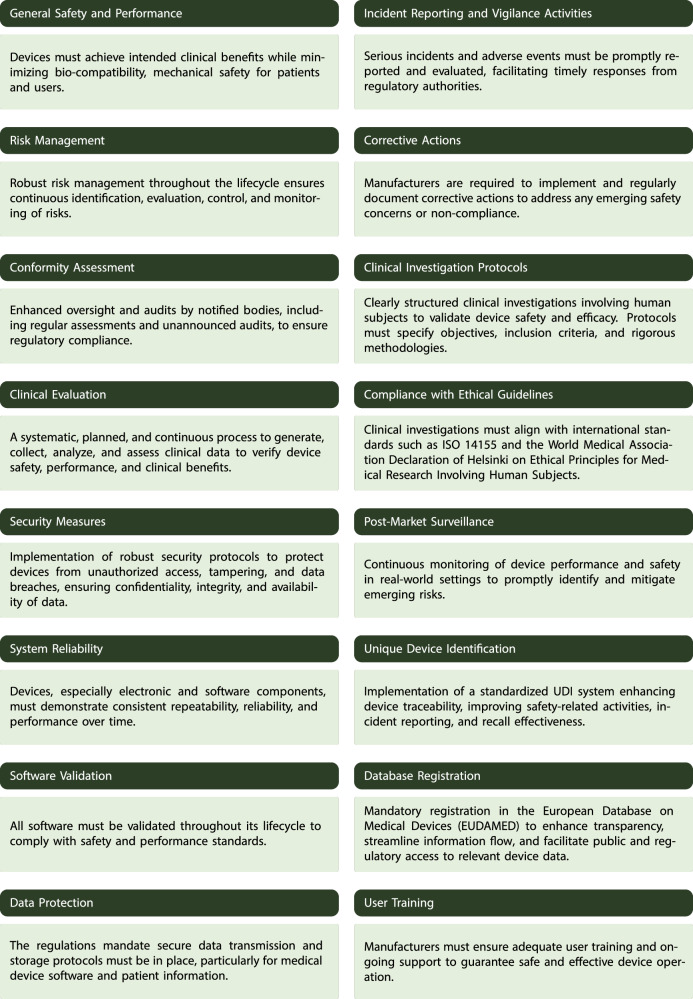
An overview of the EU MDR requirement categories, each of which addresses a critical dimension of device safety, performance, and regulatory compliance. The figure presents high-level regulatory requirement categories derived from the European Union (EU) Medical Device Regulation, which may become particularly gap-prone in the context of data-driven devices. Foundational safety is established through `General Safety and Performance', which mandates minimising risks and ensuring biocompatibility. The lifecycle approach to safety is illustrated through `Risk Management', `Corrective Actions', and `Post-Market Surveillance', ensuring continuous identification and mitigation of emerging risks. Technical robustness is addressed by `Security Measures' for data integrity, `System Reliability' for consistent performance, and `Software Validation'. Clinical evidence generation is governed by `Clinical Evaluation' and `Clinical Investigation Protocols', which must align with `Ethical Guidelines'. Administrative compliance includes `Conformity Assessment' by notified bodies, `Incident Reporting', `Unique Device Identification' (UDI) for traceability, and mandatory `Database Registration' in the European Database on Medical Devices (EUDAMED). Additionally, `Data Protection' ensures secure storage and use of patient information, while `User Training' guarantees safe device operation.

The categories of the EU MDR requirements are outlined in Fig. 2. Within the regulation, technical documentation is structured by Annex II (Sections 1-6) and Annex III. Each category in our taxonomy maps to one or more of these sections, as detailed in Table 1. Cross-cutting categories such as risk management, security measures, system reliability, software validation, and data protection are maintained as standalone contributing requirements to reflect their continuous application throughout the device lifecycle.

**Table 1:** Mapping of EU MDR technical documentation to requirement categories with respect to EU MDR Annex II (Sections 1-6) and Annex III (PMS)

EU MDR Annex II & Annex III item	EU MDR requirement category	Justification
Annex II-1 Device description and specification (incl. variants, accessories)	General safety and performance	Intended purpose, classification, design features anchor GSPR evidence.
Annex II-2 Information to be supplied by the manufacturer (labels/IFU)	Usability & user training	IFU/label content drives safe use and residual-risk controls.
Annex II-3 Design and manufacturing information	Conformity assessment readiness	Traceable design/manufacture inputs and controls.
Annex II-4 General Safety and Performance Requirements (GSPR)	General safety and performance	Direct statement of conformity to EU MDR Annex I.
Annex II-5 Benefit-risk analysis and risk management	Risk management	Documented risk file, controls, and residual-risk acceptability.
Annex II-6 Product verification & validation (pre-clinical, software V&V, clinical data)	Software validation, clinical evaluation	Objective evidence for performance and claims; software evidence placed here.
Annex III PMS plan (incl. methods, data sources, triggers)	PMS, CAPA linkages	Lifecycle surveillance, updates feeding risk and clinical evaluation.

## Review of the standards on data safety and quality assurance

The information security and privacy standards, including ISO/IEC 27001^22^, 27002^23^, 27701^24^, and 29100^25^, provide frameworks for securing personal and health-related data, establishing risk-based controls, and supporting the GDPR (General Data Protection Regulation) compliance^25,26^. The approaches to ensure the data accuracy, completeness, system reliability, and lifecycle traceability are defined by the data and software quality standards, including ISO 8000-61, ISO 25012, and IEEE 1012^27–29^. However, these standards are not harmonised for defining “good data" for clinical safety, and they are yet insufficient to address the emerging requirements for AI system development, such as transparency, accountability, fairness, and safety. A set of AI-specific standards, including ISO/IEC 5338^30^, 25059^31^, and the BS 30440 framework^32^, has been published to address the gaps in requirements for AI systems; the gap remains for medical AI systems.

The emerging AI-specific standards further emphasise key regulatory expectations for transparency, robustness, interoperability, and accountability in the development and validation of AI-enabled medical technologies. Within this context, data is recognised as a critical asset for medical devices. It could be clinical measurements, sensor readings, or other kinds of observations that are used to build evidence and make decisions, either directly or through computational methods^33^.

To ensure the safe processing of data, ISO 62304^34^ introduced a three-level risk-based approach for possible impacts on patient safety. However, ISO 62304, first published in 2006, was originally structured around traditional waterfall development models. Recognising the growing adoption of Agile methodologies in the industry, AAMI TIR45^35^ has emerged to bridge this gap. It offers a practical framework for integrating Agile practices, including iterative development, continuous testing, and adaptive planning within the regulatory context of medical device software development. It enables developers to comply with international standards like IEC 62304 and ISO 14971 while benefiting from the agility and responsiveness of modern software engineering practices.

Data safety and quality are dependent requirements, as both are vital for reliable medical decision-making. According to the European Medicines Agency (EMA) Data Quality Framework^33^, “data quality is an attribute of a product or service that defines the degree to which it meets customer and other stakeholder needs within statutory and regulatory requirements, or its fitness for intended use." Table 2 outlines key dimensions of data quality identified in the EMA framework, along with corresponding metrics for assessment. A major challenge for data quality is bias, which can arise from various factors such as population imbalances, frequency differences, or inconsistent instrumentation^36,37^. Algorithms trained on biased data may exhibit compromised performance when deployed across diverse patient populations, thus presenting potential risks to patient safety.

**Table 2:** Dimensions and metrics for assessing data quality in datasets for data-driven medical devices

Dimension	Metric	Description
Reliability	Accuracy	Degree to which data reflects the real-world values or clinical observations.
	Precision	Level of detail or granularity in the data measurements.
	Plausibility	Extent to which data appears reasonable and clinically coherent.
	Traceability	The ability to track data on how it was generated, processed, and stored.
Extensiveness	Completeness	The amount of data available concerning the total amount that could be available, given the capture process and data format.
	Coverage	The amount of information available for real-world entities it represents, such as the percentage of a given population data.
Coherence	Format Coherence	To ensure data is presented in a consistent format.
	Structural Coherence	To ensure the relationships between data elements are consistent.
	Semantic Coherence	To ensure terminology is used consistently across the data.
	Uniqueness	To ensure the same information is not duplicated in the dataset.
Timeliness	Currency	To reflect how current the data is when used.
Relevance	Relevance	To assess whether the data contains the required elements necessary to answer a specific decision-making procedure.

These criteria are critical for evaluating the suitability of data used throughout the device lifecycle, including development, validation, and monitoring.

## Gap analysis

**Fig. 3:**
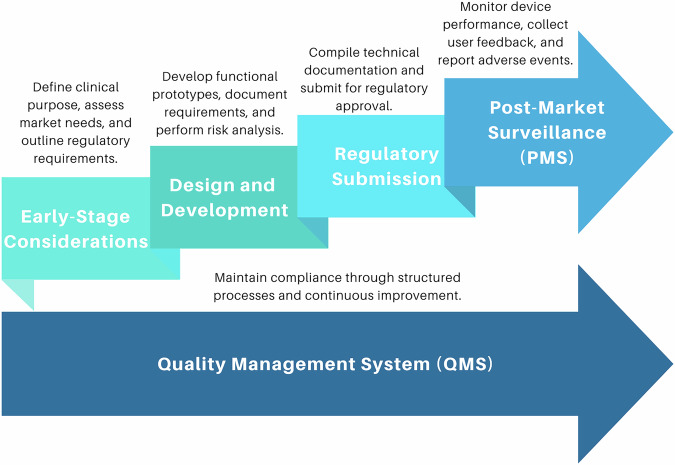
The medical device regulatory lifecycle includes several main stages. First, `Early-Stage Considerations' includes defining the clinical purpose, assessing market needs, and outlining the regulatory requirements. The next step, `Design and Development', is where functional prototypes are created, requirements are documented, and a risk analysis is conducted. The `Regulatory Submission' stage follows and involves preparing technical documentation for regulatory approval. The `Post-Market Surveillance (PMS)' stage is focused on monitoring performance, collecting user feedback, and reporting adverse events. Under these sequential steps, the `Quality Management System' (QMS) runs in the entire lifecycle for maintenance of the compliance. These stages are further mapped to the Global Harmonization Task Force (GHTF) Regulatory Model stages in the Supplementary Documents.

The regulatory lifecycle has five stages: (1) early-stage considerations, (2) design and development, (3) regulatory submission, (4) PMS, and (5) QMS^15^. This simplified frame, as in Fig. 3, is aligned with the internationally agreed Global Harmonization Task Force (GHTF) Regulatory Model, which emphasises PMS and vigilance, QMS, risk management, and regulatory auditing throughout the lifecycle but groups the activities into five steps for further clarity for technical developers (see Supplementary Material for the mapping)^38^. Table 3 maps EU MDR requirements across the regulatory lifecycle of medical device systems, aligning specific regulatory obligations with corresponding lifecycle stages, key compliance activities, and required documentation. While it is applicable to all medical devices, it includes the emerging requirements for data-driven medical devices, such as continuous validation processes, timely corrective actions, and ensuring transparency and explanation in clinical settings^39^.

**Table 3:** Mapping of EU MDR requirements across the device lifecycle

EU MDR Requir.	Lifecycle Stage	Key Actions	Required Documentation
General Safety and Performance	Entire lifecycle	Definition, validation, and continuous monitoring of intended clinical benefits	Technical documentation, GSPR checklist, Declaration of Conformity, verification and validation reports, continuous monitoring logs
Risk Management	Entire lifecycle	Identification, assessment, and ongoing mitigation of risks	Risk management file (ISO 14971), ongoing risk management review documentation
Conformity Assessment	Design, Regulatory Submission	Risk classification, preparation of conformity assessment documents	Declaration of Conformity, EU Technical Documentation Assessment Certificates, EU Type-examination Certificates
Clinical Evaluation	Design, QMS, Regulatory Submission	Clinical testing, validation	Clinical Evaluation Report (CER), Clinical investigation data (MEDDEV 2.7/1 Rev. 4, ISO 14155 compliance documents)
Security Measures	Design, QMS, PMS	Implementation of Security Measures	Security Risk Management Documentation, compliance with MDCG 2019-16, security testing reports, vulnerability assessments
System Reliability	Design, PMS	Reliability Validation and Performance Drift Monitoring	Validation test protocols, reliability assessment documents, continuous performance monitoring and drift assessment reports
Software Validation	Design, QMS	Verification and validation of software functionality	Software validation plan, validation and verification reports, software lifecycle management documents (IEC 62304 compliance)
Data Protection	Design, QMS, PMS	Implementation and verification of data protection strategies	Data protection policies, evidence of compliance with GDPR, encryption details, data security protocol documentation
Incident Reporting	PMS	Continuous monitoring, reporting of adverse events and system failures	Incident reporting protocols, incident reports, vigilance records, PSUR (Periodic Safety Update Reports)
Corrective Actions	QMS, PMS	Implementation of corrective and preventive actions (CAPA)	CAPA reports, documented procedures, results of corrective actions, verification of effectiveness of implemented actions
Clinical Investigation Protocols	Design, Regulatory submission	Structured clinical investigation to validate safety and efficacy	Clinical investigation protocols, clinical study reports compliant with ISO 14155
Compliance with Ethical Guidelines	Design, Regulatory submission, PMS	Ethical oversight of clinical studies, AI-specific ethical considerations	Ethical compliance documentation, adherence to Declaration of Helsinki, ISO 14155 compliance, ethical review board approvals
PMS	PMS	Continuous real-world performance monitoring, feedback incorporation	PMS plan, PMS reports, continuous monitoring logs, PSUR
UDI	Regulatory Submission, PMS	Assignment and traceability of Unique Device Identification	UDI documentation, registration in EUDAMED
Database Registration	Regulatory submission	Registration of device information	EUDAMED registration documentation, EU declaration of conformity
User Training	PMS	Training healthcare providers and end-users	Training materials, user manuals, ongoing user feedback, and update logs

For each requirement, it outlines the relevant lifecycle phase(s), the critical compliance activities to be performed, and the types of documentation expected to demonstrate conformity.

Existing regulatory oversight mechanisms need to evolve to accommodate the dynamic, adaptive, and data-driven characteristics of AI technologies to ensure safe use. Within the broader shift toward data-driven systems, synthetic data can emerge as a key enabler of developments in healthcare due to its capabilities for augmenting real-world datasets while addressing privacy and availability constraints. Yet, concerns over authenticity, fidelity, and evidentiary reliability limit its regulatory acceptance. As noted in the Global Summit on Regulatory Science 2024^40^, robust validation protocols, transparency, and traceability mechanisms are essential to building trust in such technologies. For clarity, these gaps are organised into thematic categories aligned with regulatory lifecycle stages, as summarised in the Table 4 and detailed in the subsequent subsections.

**Table 4:** Regulatory gaps for data-driven medical devices with respect to EU MDR references and corresponding standards and guidance

Gap	EU MDR Reference	Relevant Standards/Guidance	Relevance to Gap
Data quality and bias mitigation	Annex I (GSPR 17.1), Annex II (6.1)	ISO 8000-61, ISO/IEC 25012, ISO/IEC TR 24027, IEEE 7003, ISO/IEC 25059	Partially support the MDR requirements on data quality and bias, but none is tailored to medical devices.
Transparency and explainability	Annex I (17.2), Annex II (6.1), Annex XIV	ISO/IEC 25059, ISO/IEC TR 24028, IEEE 7001, BS 30440, ISO/IEC 12792	Supporting measurable transparency/explainability controls and generating MDR technical evidence.
External validation and generalisability	Annex I (GSPR 17.2), Annex XIV	BS 30440, ISO/IEC 5338, IEEE 1012, ISO/IEC 25059	Support structure, planning, execution and evidence of external validation, but need to be integrated with MDR Annex XIV.
Standardised AI performance metrics	Annex I (GSPR 17.2), Annex XIV	ISO/IEC 25059, ISO/IEC TS 25058, ISO/IEC 5338, BS 30440	Supports what to measure, how to measure/evaluate, where to place metrics in the AI lifecycle, and BS 30440 ties metrics to healthcare validation.
Risk management for AI failure	Annex I (3), Article 10(2)	ISO 14971, ISO/IEC 23894, ISO/TR 24971, ISO/IEC TR 24029-1, IEC 62304, ISO/TS 24971-2	Supports AI-specific risk sources, treatments, and robustness methods to verify controls; ISO/IEC 23894 and ISO/IEC TR 24029-1 are not medically tailored.
Consideration of edge cases	Annex I (17.2), Annex XIV	ISO/IEC TR 24029-1, ISO/IEC 42005, BS 30440	Supports identification and testing of edge cases but is not tailored for medical use.
Clinical evaluation for AI	Article 61, Annex XIV	ISO 14155, BS 30440, MDCG 2020-1, IMDRF N41	Supports how to generate evidence for AI claims.
Human-AI collaboration	Annex I (1), Annex XIV	IEEE Ethically Aligned Design, ISO/IEC 12792, ISO/IEC 25059, BS 30440	Supports the definition of human-centred controls and quality attributes.
Use of synthetic data	Annex XIV (1), Annex II (6.1)	ISO/IEC JTC 1/SC 42, BS 30440	Provides an overview of synthetic data concepts and methods, but not development to replace clinical evidence.
Continuous-learning AI	Article 83, Annex III	ISO/IEC 5338, AAMI CR510, IMDRF ML Framework, ISO/TR 20416	Support the governance of continuous learning post-market, but not tailored for medical context.
Change management	Annex III, Annex IX	ISO/TR 20416, IEC 62304, ISO 13485, ISO/IEC 5338	Support the EU MDR Annex III documentation set and Annex IX conformity assessment.
PMS for AI drift	Article 83, Annex III	ISO/TR 20416, ISO/IEC 5338, AAMI CR510, IMDRF N60	Supports trustworthy cloud pipeline, lifecycle cybersecurity, but not specified for capturing drifts during PMS.
AI-specific security	Annex I (17.2), Annex XIII	MDCG 2019-16, ISO/IEC 27001, ISO/IEC 23894, IEC 81001-5-1	Supports technical documentation for design, risk files and PMS procedures.
Harmonized standards	Annex I (1)	IMDRF GMLP, ISO/IEC 25059, ISO 14971, IEC 62304	Support global principles, not EU-harmonized, except ISO 14971.
Classification uncertainty	Annex VIII (Rule 11)	MDCG 2019-11, ISO/IEC 5339, ISO/IEC 23894	Not sufficient support for classify medical risk.
AI ethics in healthcare	Annex I (1)	IEEE Ethically Aligned Design, ISO/IEC 42005, PD IEC SRD 63416	Supports ethics considerations in design.
Cross-border data sharing	Article 10, GDPR	ISO/IEC 27701, ISO/IEC 27018	Support techniques could be used for cross-border data sharing.
Interoperability	Annex I (17.2), Article 10(9)	IEEE P2933, ISO/IEC 27001, HL7 FHIR, IEC 62304	Supports the specification, governance and engineering of interoperability.

### Data requirements and algorithm transparency

#### Data quality and bias mitigation

Current regulations do not have specific requirements and guidance for data quality, diversity, and bias mitigation for training AI algorithms. This gap is critical for manufacturers to ensure safety and performance in AI systems by explicitly addressing fairness issues (e.g., performance across demographics), which can lead to unaddressed bias or reduced effectiveness in certain patient subgroups^9^.

#### Data availability and quality assurance

Data-driven devices require vast amounts of high-quality data, yet there is no mechanism to ensure the availability of quality data for development. There is a regulatory gap in guidance on acquiring and curating real-world data for model training and validation, which can cause delays in development or lead to reliance on limited datasets. Schwabe et al. proposed a framework for dataset quality assessment and highlighted the gaps in the current regulatory framework for assessing bias in the training datasets^41^.

#### Transparency and explainability of AI decisions

The regulation does not mandate transparency or explainability for “black-box” AI models. There is no guideline to support healthcare practitioners in interpreting AI decision-making processes for patient safety^41,42^.

### Performance validation standards and risk controls

#### Guidance on external validation and generalisability

EU MDR requires evidence of safety and performance, yet there is a gap for specific guidelines for validating AI models on external, independent datasets. AI algorithms may have been tested only on training data, which can lead to overestimating their performance^43^.

#### Standardised metrics for AI performance and safety

There is no standardised set of performance metrics or benchmarks for data-driven medical devices. It includes comparisons and regulatory evaluations for algorithmic transparency, accuracy, and reliability in clinical conditions^15,41^.

#### Risk management for AI failure modes

Traditional medical devices’ general safety and risk management requirements may not fully cover AI-specific failure modes, such as algorithmic bias, explainability issues, and performance drift.^41,44^. There is a gap in guidance on identifying and controlling risks throughout the lifecycle of medical devices containing data-driven components.

#### Consideration of edge cases

The current framework does not explicitly require testing for edge cases or foreseeable misuse cases^45,46^. For example, a data-driven dermatology diagnostic tool may underperform for an under-represented group of patients (such as certain skin tones). Yet, there is no validation framework covering such scenarios, which may lead to devices entering the market without fully understanding their limitations.

### Clinical evaluation and evidence generation

#### Clinical evaluation requirements of data-driven medical devices

The current regulation mandates clinical evaluation, but it does not offer sufficient direction for data-driven devices. In silico evaluations (i.e., retrospective, on datasets) are commonly used, but the data-driven devices would benefit from clinical evaluations, which could be helpful in understanding how AI devices perform in practice. However, there are no standards for ‘sufficient clinical evidence’. Traditional metrics such as model accuracy or AUC (area under the curve) are unable to directly translate into evidence of clinical outcomes or safety. There is a need for prospective, human-in-the-loop, and randomised trials to assess how AI impacts patient care and clinicians’ decisions^47^.

#### Guidance on human-AI collaboration in clinical settings

Data-driven systems are increasingly being integrated into clinical decision-making processes^48^. Still, there is insufficient regulatory guidance on how human clinicians should interact with AI systems, particularly in disagreement or conflicting recommendations. There is a gap in defining the role of healthcare professionals’ oversight when AI is used to support diagnosis, treatment decisions, or other critical healthcare functions. The IEEE Ethically Aligned Design guideline supports human-in-the-loop approaches but is not integrated into medical device regulations^49^. A new multidimensional risk matrix could be introduced that goes beyond traditional consequence-and-likelihood models. It can include a third axis focused on data-centric statistical scenarios, such as cohort sizes in population screening, variability in data quality, or confidence intervals in model predictions.

#### Guidelines for using synthetic data for evidence generation

Current regulations provide limited guidance on using synthetic data for generating evidence in data-driven medical devices. Alloza et al.^50^ and Giuffre and Shung^51^ highlight the increasing role of synthetic data in regulatory decision-making due to its ability to supplement real-world data and address privacy concerns. However, there is a gap in how regulators evaluate synthetic data quality, reliability, and limitations. Guidelines are needed to ensure that synthetic data used for training AI models is validated and comparable to real-world data, ensuring its appropriateness for evidentiary support in regulatory submissions.

### Adaptive algorithms and long-term performance

#### Regulating continuous-learning AI algorithms

The current regulation is designed for static devices at the time of certification. However, some AI systems can be classified as adaptive AI, which refers to the systems able to continuously learn based on new data at runtime to adapt more quickly to changes in real-world circumstances by updating themselves in real time or via periodic retraining^52^. Although PMS and clinical follow-up frameworks are established, there is no explicit regulatory provision for approving or monitoring self-learning algorithms that may change after deployment^15,42^. This gap may cause manufacturers to either freeze AI models (i.e., lock the algorithm), which may hinder innovation.

#### Change management and update requirements

The current regulation does not detail how learning-based software updates (e.g., to fix biases or improve accuracy) should be handled from a regulatory standpoint. Any significant algorithm change requires a new conformity assessment, and such a mechanism needs to be included in PMS plans^42,53,54^.

#### Regulatory adaptation for PMS

Although EU MDR strengthened PMS obligations, it does not provide details for monitoring of AI algorithms for performance drift or degradation over time^15,55,56^. Data-driven devices may become less effective if patient demographics or usage conditions shift. There is a need to establish mechanisms for manufacturers to modify and revalidate the data-driven system in response to performance variations detected in the post-market phase^57^.

#### AI-specific cybersecurity risks

There are guidelines for general IT security risks, risk management, and PMS for medical devices. However, there is a gap in addressing data-driven attacks, such as adversarial machine learning attacks, dataset poisoning, or exploiting vulnerabilities in AI algorithms^44,58^. There is a necessity for developing guidelines for AI-specific risks, such as expanding upon MDCG 2019-16^59^ by addressing data poisoning, model inversion attacks, and adversarial machine learning attacks^60,61^. It is also required to introduce post-market monitoring frameworks for detecting anomalous behaviours and security breaches in real-time and ensuring data-driven medical devices remain secure over time.

### Regulatory guidance

#### Harmonized standards and specific guidelines

There is a lack of coordination between regulatory bodies (e.g., EU, FDA, TGA, and MHRA) for data-driven medical devices that are evaluated and approved globally. While manufacturers often rely on general software engineering standards, initiatives from bodies such as IMDRF are crucial for bridging these gaps. The IMDRF plays a pivotal role in accelerating international regulatory convergence by fostering dialogue among regulators from major markets. Despite these efforts, a unified international standard for critical aspects, such as definitions, risk management strategies, algorithm bias, performance validation, or explainability is still lacking. Such a lack of uniformity creates a complex regulatory landscape for manufacturers to navigate multiple regulatory jurisdictions^55^.

Furthermore, the EU AI Act is published as the world’s first legal framework for AI. It is applicable alongside the EU MDR for AI-enabled devices that are generally classified as “high-risk” if the device is subject to a third-party conformity assessment by a notified body. AIB 2025-1/MDCG 2025-6 was released to clarify the interface between these documents and confirmed that the AI Act is a complementary layer, adding AI-specific requirements which are not explicitly detailed in the EU MDR^62^. The AI Act relies on standards to provide a ‘presumption of conformity’ to its legal requirements. While the foundational software lifecycle (IEC 62304)^34^ and risk management (ISO 14971)^18^ standards for medical devices remain applicable, the standards ISO/IEC 42001^63^ and ISO/IEC 23894^64^ provide requirements for an AI management system and for AI risk management, respectively, although they do not specifically address AI in the field of medical devices. Of note, the new specification ISO/TS 24971-2 “Medical devices - Guidance on the application of ISO 14971 - Part 2: Machine learning in artificial intelligence" has been developed to interpret ISO 14971 in the context of AI-enabled medical devices. ISO/TS 24971-2 is currently ahead of publication^65^.

#### Classification uncertainty

Rule 11 of the EU MDR defines the classification rules of medical devices. However, there is a lack of clarity to determine the risk category for data-driven systems^15^. There is a need for developing risk classification frameworks that reflect the risks posed by data-driven systems, including classification tools such as decision trees to guide the manufacturers in assessing risk levels.

#### Guidelines for AI ethics in healthcare

Frameworks such as the EU AI Act^66^ emphasise the need for ethical AI, but they are not fully integrated into medical device regulations. Current regulatory frameworks focus on technical safety, efficacy, and performance but do not adequately address the ethical considerations for data-driven systems. These considerations include the issues of autonomy, accountability, and patient consent, mainly when data-driven systems make clinical decisions. While the technical requirements are discussed more, less focus is given to the ethical implications of data-driven decision-making processes.

#### Guidance for cross-border data sharing

Data-driven medical devices often use data for improvement. Such data may be collected through the real-world usage of the device, which would include cross-border data sharing. Still, current regulations do not clarify how such data can be shared and used across jurisdictions, and privacy regulations such as GDPR in Europe limit data availability for training AI models^67,68^.

#### Interoperability of data-driven systems with the legacy systems

The existing regulations provide guidance for IT security and integration, but there is a need for guidance on the interoperability of data-driven systems with the existing healthcare infrastructure, which often consists of non-data-driven systems^69^. It is also linked to regulatory adaptation for PMS and global harmonisation gaps. To ensure the integration of data-driven systems with legacy medical and IT systems, interoperability guidelines promoting standard communication protocols and interfaces must be developed.

## Conclusion

The existing framework for governing the safety and quality assurance of medical devices does not fully cover the evolving requirements of the industry due to the increasing use and benefits of data-driven assets in medical devices. To address these gaps, the study identifies five key recommendations for regulatory and industry stakeholders.

First, the development of real-time compliance mechanisms is crucial to support continuous post-market surveillance and enable the timely detection of performance drift in adaptive algorithms. Second, establishing standardised protocols for data quality and bias mitigation is essential to ensure the robustness, fairness, and safety of AI models across diverse patient populations. Third, greater global regulatory harmonisation is necessary to simplify cross-border compliance, reduce duplication of effort, and enable the secure exchange of data and evidence. Fourth, transparency and explanation should be formalised within regulatory frameworks to promote ethical accountability and foster trust between clinicians and patients in AI-assisted decision-making. Finally, tailored cybersecurity standards for data-driven systems are needed to address unique vulnerabilities, such as adversarial attacks and model manipulation, and to safeguard both patient data and clinical outcomes. By advancing these targeted recommendations, this study aims to support the evolution of an agile, risk-aware, and globally harmonised regulatory landscape better suited to data-driven medical devices.

## Supplementary information

Supplementary information

## Data Availability

No datasets were generated or analysed during the current study.
