# Supplementary material for: Data-driven medical devices and the EU MDR: mapping gaps in standards for regulatory compliance
Source: Npj Health Syst. 2026 Mar 9;3:21. doi: 10.1038/s44401-026-00075-2 (PMC13354248; doi:10.1038/s44401-026-00075-2)
Supplement: Supplementary file 1 — Supplementary information [file 44401_2026_75_MOESM1_ESM.pdf]

Supplementary Material

1 Mapping Regulatory lifecycle to the GHTF Regulatory Model

This section aims to clarify how the considered five-stage lifecycle corresponds to the harmonised GHTF Regulatory Model. The Global Harmonization Task Force (GHTF) depicts the device lifecycle with the stages begining from *concept* through *product realisation* to *placing on the market*, *product use*, and *end of product life*, with *QMS*, *risk management*, and *complience auditing* applied across the stages of the regulatory model in the GHTF/AHWG-GRM/N1R13:2011.

Table S1: Regulatory lifecycle stages with respect to GHTF Regulatory Model Stage

**Regulatory Lifecycle Stage**	**GHTF Regulatory Model Stage**	**Alignment note**
Early-stage considerations	Concept	Define intended purpose, clinical context and classification, principles and applicable standards, high-level risk profile and assumptions.
Design and development	Product realisation, Pre-market regulatory controls	Design control, V&V , supplier and process validation, risk management, technical documentation and pre-market evidence.
Regulatory submission	Placing on the market	Conformity assessment, notified-body review, CE marking, market placement.
Post-market surveillance (PMS)	Product use, End of Product Life	PMS/vigilance, Post-Market Clinical Follow-up, CAPA, real-world performance monitoring, servicing, decommissioning and retirement controls.
Quality management system (QMS) *(cross-cutting stage)*	QMS, Risk management, Compliance auditing *(cross-cutting stages)*	ISO 13485-orientated QMS, ISO 14971-orientated risk management, change control and regulatory audit.

2 Lists of Reviewed Standards & Guidance Documents

Table S2: List of healthcare data, quality and lifecycle standards.

#	**Title of the document**	**Pub.**	**EU-Har.**
1	ISO 13485:2016 Medical devices — Quality management systems — Requirements for regulatory purposes	✓	✓
2	ISO 14971:2019 Medical devices — Application of risk management to medical devices	✓	✓
3	ISO/TR 24971:2020 — Medical devices—Guidance on the application of ISO 14971	✓	×
4	ISO/TR 20416:2020 — Medical devices—Post-market surveillance for manufacturers	✓	×
5	IEC 62304:2006 — Software life cycle processes	✓	✓
6	ISO/IEC 82304-1:2016 — Health software—Part 1: General requirements for product safety	✓	×
7	ISO/TS 82304-2:2021 — Health software—Quality and reliability criteria for health and wellness apps	✓	×
8	IEC/TR 80002-1:2009 — Medical device software—Guidance on applying ISO 14971 to software	✓	×
9	ISO/TR 80002-2:2017 — Medical device software—Validation of software for QMS	✓	×
10	IEC/TR 80002-3:2014 — Process reference model for IEC 62304	✓	×
11	AAMI TIR45:2023 — Guidance on agile practices in medical device software	✓	×
12	IEC 62366-1:2015 Medical devices Part 1: Application of usability engineering to medical devices	✓	×
13	ISO 27799:2016 — Health informatics—Information security management in health using ISO/IEC 27002	✓	×
14	ISO 20417:2021 — Medical devices—Information to be supplied by the manufacturer	✓	×
15	ISO 14155:2020 — Clinical investigation of medical devices for human subjects — Good clinical practice	✓	×
16	IEC 80001-1:2021 — Application of risk management for IT-networks incorporating medical devices	✓	×
17	IEEE 11073-10700-2022 - IEEE Standard – Health Informatics–Device Interoperability Part 10700: Point-of-Care Medical Device Communication–Standard for Base Requirements for Participants in a Service-Oriented Device Connectivity (SDC) System	✓	×
18	IEEE 2802:2022 — Performance and safety evaluation of data-driven medical devices	✓	×
19	ISO 15189:2022 — Medical laboratories—Requirements for quality and competence	✓	×
20	ISO/IEC 17025:2017 — General requirements for competence of testing and calibration laboratories	✓	×
21	ISO 18308:2011 — Health informatics—Requirements for an electronic health record architecture	✓	×
22	HL7 EHR-S Functional Model, Release 2.1	✓	×
23	HL7 FHIR — Fast Healthcare Interoperability Resources	✓	×
24	HL7 FHIR Implementation Guide: Personal Health Device (PHD IG)	✓	×
25	HL7 FHIR Implementation Guide: Point-of-Care Device (PoCD IG)	✓	×
26	IHE Devices (DEV) — Service-oriented Device Point-of-care Interoperability (SDPi)	✓	×
27	IEEE/UL 2933:2024 — Clinical IoT Data and Device Interoperability with TIPPSS	✓	×
28	OMOP Common Data Model (CDM) — Observational Medical Outcomes Partnership	✓	×

Table S3: List of Security & privacy standards, EU/UK and guidance documents

#	**Title of the document**	**Pub.**	**EU-Har.**
29	ISO/IEC 27001:2023 — Information Security Management Systems (ISMS) Requirements	✓	×
30	ISO/IEC 27002:2022 — Information security, cybersecurity and privacy controls	✓	×
31	ISO/IEC 27018:2020 — Protection of PII in public clouds	✓	×
32	ISO/IEC 29100:2020 — Privacy framework	✓	×
33	ISO/IEC 29151:2022 — Code of practice for PII protection	✓	×
34	ISO/IEC 27701:2021 — Privacy Information Management	✓	×
35	GDPR (EU 2016/679) — General Data Protection Regulation	✓	✓
36	ENISA (2013–2022) — Big Data Threat Landscape; Big Data Security; Privacy by Design in Big Data; Personal Data Clouds; Data Protection Engineering	✓	✓
37	EMA/326985/2023 — Data Quality Framework for EU medicines regulation	✓	✓
38	MEDDEV 2.7/1 rev.4 — Clinical Evaluation	✓	✓
39	MDCG 2019-11 — Qualification and classification of software (MDR/IVDR)	✓	✓
40	MDCG 2019-16 — Guidance on cybersecurity for medical devices	✓	✓
41	MDCG 2020-1 — Clinical/Performance evaluation of medical device software	✓	✓
42	MDCG 2020-5 — Clinical evaluation—Equivalence	✓	✓
43	MDCG 2021-24 — Guidance on classification of medical devices	✓	✓
44	MHRA (2024) — Software and artificial intelligence (AI) as a medical device: guidance	✓	×

Table S4: List of AI governance, AI quality and IMDRF documents.

#	**Title of the document**	**Pub.**	**EU-Har.**
45	ISO/IEC 42001:2023 — Management system standard for AI (AIMS)	✓	×
46	ISO/IEC 12792 — AI Transparency Taxonomy (under publication)	×	×
47	ISO/IEC 42005:2025 — AI System Impact Assessment	✓	×
48	ISO/IEC 5338:2023 — AI system life-cycle processes	✓	×
49	ISO/IEC 5339:2024 — Guidance for AI applications	✓	×
50	ISO/IEC TR 24027:2021 — Bias in AI systems and AI-aided decision making	✓	×
51	ISO/IEC TR 24028:2020 — Overview of trustworthiness in AI	✓	×
52	ISO/IEC TR 24029-1:2021 — Artificial Intelligence (AI) — Assessment of the robustness of neural networks	✓	×
53	ISO/IEC 24029-2:2023 — Assessment of robustness of neural networks—Formal methods	✓	×
54	ISO/IEC TR 24030:2024 — AI use cases	✓	×
55	IEC SRD 63416:2023 — Ethical considerations of AI in Active Assisted Living	✓	×
56	BS 30440:2023 — Validation framework for AI in healthcare	✓	×
57	EU AI Act— Regulation on Artificial Intelligence	✓	✓
58	NIST AI Risk Management Framework	✓	×
59	Singapore AI Verify — AI Governance Testing Framework and toolkit	✓	×
60	IMDRF/SaMD WG/N12 — Software as a Medical Device: Risk categorization framework	✓	×
61	IMDRF/SaMD WG/N23 — SaMD: Application of Quality Management System	✓	×
62	IMDRF/SaMD WG/N41 — SaMD: Clinical Evaluation	✓	×
63	IMDRF/Standards WG/N51 — Optimizing standards for regulatory use	✓	×
64	IMDRF/GRRP WG/N52 — Principles of labelling for MD and IVD MD	✓	×
65	IMDRF/MDCE WG/N56 — Clinical Evaluation	✓	×
66	IMDRF/MDCE WG/N57 — Clinical Investigation	✓	×
67	IMDRF/PMD WG/N58 — Personalized Medical Devices—Regulatory pathways	✓	×
68	IMDRF/CYBER WG/N60 — Principles and practices for MD cybersecurity	✓	×
69	IMDRF/GRRP WG/N66 — Recognition of Conformity Assessment Bodies	✓	×
70	IMDRF/AIMD WG/N67 — ML-enabled Medical Devices: Key terms and definitions	✓	×
71	IMDRF/CYBER WG/N70 — Cybersecurity of legacy medical devices	✓	×
72	IMDRF/GRRP WG/N71 — Medical Device Regulatory Review Report: Information to be included	✓	×
73	IMDRF/CYBER WG/N73 — Software Bill of Materials for medical device cybersecurity	✓	×
74	IMDRF/PMD WG/N74 — Personalized Medical Devices—Production verification and validation	✓	×
75	ISO/IEC 25059:2024 — SQuaRE—Quality model for AI systems	✓	×
76	ISO/IEC TS 25058:2024 — SQuaRE—Guidance for quality evaluation of AI systems	✓	×
